# Accelerating achievement for Africa’s adolescents

**Published:** 2025-10-07

**Authors:** Lorraine Sherr, Lucie Cluver, Chris Desmond, Mark Orkin, William Rudgard, Elona Toska, Olayinka Omigbodun, Marisa Casale, Kate Orkin, Charles Falajiki, Evelyn Gitau, Henriques Andela, Heidi Stöckl, Elleke Boehmer, Douglas Webb, Louise Gordon, Mandeep Dhaliwal, Franziska Meinck

**Affiliations:** a Institute for Global Health, University College London, London, UK;; b Department of Social Policy and Intervention, University of Oxford, Oxford, UK;; c Department of Child and Adolescent Psychiatry, University of Cape Town, Cape Town, South Africa;; d Centre for Rural Health, University of KwaZulu-Natal, Durban, South Africa;; e MRC/Wits Developmental Pathways for Health Research Unit, School of Clinical Medicine, University of the Witwatersrand, Johannesburg, South Africa;; f Centre for Social Science Research, University of Cape Town, Cape Town, South Africa;; g Centre for Child and Adolescent Mental Health, College of Medicine, University of Ibadan, Ibadan, Nigeria;; h School of Public Health, University of the Western Cape, Cape Town, South Africa;; i Blavatnik School of Government, University of Oxford, Oxford, UK;; j Department of Education, University of Oxford, Oxford, UK;; k Aid for Rural Education Access Initiative, Abuja, Nigeria;; l Science for Africa Foundation, Nairobi, Kenya;; m Faculty of Medicine, Eduardo Mondlane University, Maputo, Mozambique;; n Institute for Medical Information Processing, Biometry and Epidemiology, Faculty of Medicine, Ludwig-Maximilians-Universität München, Munich, Germany;; o Faculty of English, University of Oxford, Oxford, UK;; p United Nations Development Programme, Tbilisi, Georgia;; q United Nations Development Programme, New York, NY, USA;; r School of Social and Political Science, University of Edinburgh, Edinburgh, UK;; s School of Public Health, University of the Witwatersrand, Johannesburg, South Africa;; t Optentia, Faculty of Health Sciences, North-West University, Vanderbijlpark, South Africa

**Keywords:** Adolescents, Africa, accelerators, health, education, violence

## Abstract

By 2050, one in four people in the world will live in Africa. The region’s adolescents are our present and future but need effective services to support their holistic health and wellbeing, advance their education, allow them to flourish, and protect them from adversity. This paper reviews evidence for a new concept of ‘development accelerators’: interventions that can help achieve multiple Sustainable Development Goals simultaneously. Across 26 studies, the most frequently identified accelerators were government-provided social protection, positive parenting including good supervision of their adolescents, adolescent-friendly healthcare and childcare, positive gender norms, and psychosocial support. These have been shown to be highly cost-effective, giving substantial returns on investment, especially when applied in combination.

## Introduction

By 2050, Africa will be home to half a billion adolescents aged 10–19 (UNDESA, 2022), experiencing rapid physical, social, emotional, and cognitive development. They bring immense dynamism and promise, but also acute sensitivity to adversity (Patton et al., 2016). Often discussed as a future generation, they are also a present generation, deserving of the most effective services.

However, in the rush to address the region’s overall needs, adolescents are repeatedly overlooked (Slogrove et al., 2017). They are neither infants nor children, nor yet adults. But Africa’s adolescents lead on experiencing negative social and health impacts: high rates of hunger, HIV infection, early pregnancy, education loss, and mental health distress (Christian & Smith, 2018; Roberts et al., 2021; Shorey et al., 2022; Toska, Laurenzi et al., 2020) Adolescents are hidden in aggregated data, typically categorised as either children or adults. For example, adolescent mothers are counted in data and health services as adults, despite having very different experiences and outcomes from adult mothers (Toska et al., 2022). Where disaggregated, they are often clustered with youth up to age 30, although there are distinct differences in risks, mechanisms, and outcomes within this wide age-range. In consequence, research, policy, and programming often ignore the unique time when a young person is navigating independence, future, education, employment, and mental health concerns.

The UKRI Hub examining Accelerators for Adolescents in Africa (The Hub) was a 5-year programme set up to provide evidence based intervention policy to support this neglected group. The initiative comprised eleven African and three UK universities, in partnership with the UN Development Programme, UNICEF, and the World Health Organization. The concept of the Hub arose from discussions between a team of interdisciplinary researchers and senior government officials and UN country offices in Eswatini, Kenya, Namibia, Nigeria, South Africa, Tanzania and Zimbabwe, and consultations with adolescent advisory groups in South Africa, Sierra Leone, and Uganda. These identified three fundamental gaps in research and donor priorities.

The first gap was in scale. Where services are provided for adolescents in the region, they are often focused on delineated geographical areas or distinct sub-groups. However, policies and services that benefit adolescents should be on a population level (Hillis et al., 2016; Patel et al., 2007). Research typically did not recognise the limited financial or human resource capacity to deliver programmes at scale in low-income settings. One government minister said, ‘I just need to know what two services to deliver at a national scale’.

The second gap was in scope. Where programmes exist, they often focus on single outcomes such as HIV prevention or education – each of key importance but attending to only one dimension of an adolescent’s needs, not to the adolescent as a whole. When asked to identify their priorities, adolescents highlighted aspirations across a range of domains: they wanted to be happy and healthy, to have the means to a livelihood and a home and a family, and to live in a safe environment (Boehmer et al., 2022; Gittings et al., 2022). Policymakers identified a fundamental gap between research – which was primarily focused on single outcomes aligned with academic and professional disciplines – and their policy challenges in meeting the multiple and interlinked needs of adolescent populations. This often mirrored divisions within funding – with agencies or teams focused on single objectives – and within government structures themselves.

The third gap was in matching evidence to resources. Policymakers expressed a need to know which programmes and policies would provide the greatest value for money and could be delivered in contexts of limited human and financial resources. A prescient South African policy leader said, ‘what I need is bang for buck’. A challenge-by-challenge research approach was, thus, failing to address the holistic needs of both policymakers and adolescents themselves.

In the wider world, the Millennium Development Goals (2000–2015) were making way for the Sustainable Development Goals (SDGs) (2015–2030). The SDGs recognise both the need for holistic responses and what Achim Steiner, head of the UN Development Programme, calls ‘a declaration of interdependence’, reflecting the interlinkages of the varied strands of adolescent experience. In 2017, the UN Development Programme posited an idea of ‘development accelerators’ – programmes or interventions with simultaneous impacts across multiple SDG goals and targets (UNDP, 2023). Government social protection (often in the form of cash transfers) had been shown to be an accelerator, with multiple beneficial effects on health, education, and income generation (Remme et al., 2014). Medical interventions, such as vaccines and tobacco control, had been shown to improve not only health, but also labour market outcomes.

But there was little evidence of whether other accelerators existed, or what they were. It was on this topic the Hub set out to address. Applying the concept of development accelerators to real-world provisions for African adolescents aims to shift from an approach that asks: ‘How best to improve a wide range of individual outcomes?’, to ‘How to identify and steer common antecedents of multiple beneficial outcomes?’ This concept aimed to move priority-setting from a process of sifting through a multiplicity of outcome-specific intervention options, to identifying manageable packages of key accelerators, each bearing on several outcomes, and feasible to implement even within tightly constrained resources. This study presents a review of this emerging data base to provide an overview of findings on accelerator based studies and to evaluate the programme mechanism within which it operated.

This paper summarises the findings of the Hub. Moreover, it seeks to reflect on the initiative itself, describing the activities undertaken and the opportunities created by the collective efforts of a wide array of participants.

## Methods

This report involves two elements of appraisal to cover the Adolescent Accelerator Hub findings. Firstly a programme review was conducted to summarise the accelerator findings and secondly an reflective review was conducted, to critically examine the initiative that produced the accelerator findings.

Over the course of the five year initiative, 274 peer reviewed publications emerged. A search of these was conducted to include all studies reporting on accelerator findings. The details of the accelerator methodology and the study findings were to be reviewed.

The accelerator model aimed to test different combinations of provisions on multiple simultaneous adolescent outcomes across a range of countries in Africa. Using the key words of ‘accelerator, Sustainable Development Goals (SDG), adolescent outcomes’, we generated a set of 26 studies to be included in the overview. The 26 studies conducted across Africa gathered from all studies conducted under the GCRF Adolescent hub represents 10% of all publications. As a check to general research, the same keyword search was conducted on PubMed. This generated 53 results in total, 40 excluded for relevance, and 13 remaining all overlapping with the Accelerator Hub publications.

The studies were set up to investigate potential components and outcomes of accelerators, using existing large-scale datasets that contained variables that had potential to benefit multiple SDG goals or targets. These included both nationally representative cross-sectional datasets, such as UNICEF’s Multiple Indicator Cluster Surveys, and geographically focused longitudinal studies across the region. This included a study of 1063 adolescents living with HIV in the Eastern Cape province of South Africa, who were followed up with 94% retention after eighteen months. For potential accelerators, the studies employed standardised methodology. For all the data allowed for identification of six real-world social services ideally measured on at least two occasions with a baseline and follow-up reading. To explore impact, eleven adolescent outcomes were identified that were aligned to, or proxies for, targets within four SDGs. Finally, there was a need to control for covariates. For each study, all these variables were included in a multivariable structural equation model that allowed for correlations among outcomes. All paths were retained for comparability across outcomes. The Benjamini-Hochberg method was used to correct for a multiplicity of tests, and each outcome was checked for collinearity (Cluver et al., 2019).

Third, in order to test accelerators in a range of non-randomised survey datasets, we used generalised estimating equations (GEE) to account for the correlations between multiple outcomes (Wilson et al., 2020), and adjusted of *p*-values to account for multiple hypothesis testing (Cluver, Rudgard, et al. 2022). We prioritised longitudinal data to evaluate within-individual associations between adolescent accelerators and outcomes (Rudgard, Saminathen, et al. 2023), and used propensity score analysis for pragmatic evaluations of existing services using survey data (Figure 2) (Cluver et al., 2024; Rudgard et al., 2022).

Fourth, we aimed to incorporate inputs across social science, humanities and other disciplines, and beyond academia. A structured series of innovation and mechanism workshops aimed to generate collaborative thinking, including narrative approaches to identity construction, with attentiveness to Black consciousness and Global South leadership. We conducted exploratory work on storytelling approaches.

Fifth, we co-created research design with adolescent advisory groups in Southern, Eastern, and Western Africa. We reviewed acceptability data from adolescents and youth to develop a new framework to conceptualise and assess acceptability of policies and interventions (Casale et al., 2023; Somefun et al., 2021), and then started applying it with young people in South Africa, Nigeria, and Zambia. During the COVID-19 pandemic, we developed methods for remote adolescent engagement, utilising social media whilst retaining privacy and ethical approaches (Gittings et al., 2021), and aimed to support policy engagement to include the voices of adolescents even during the pandemic (Boehmer et al., 2021).

Sixth, we conducted two new randomised trials in order to test potential ‘accelerator’ combinations, and to develop low-cost delivery mechanisms for use in the region. These included (i) a trial with 400 villages in Kenya of cash transfers and psychosocial support – a motivational video and a long-term planning exercise (Orkin, 2022) (ii); In Tanzania, we combined an evidence-based parenting programme (Cluver et al., 2018) with evidence-based sexual violence prevention content (Decker et al., 2018; Ligiero et al., 2019), bereavement mental health support (Sherr, 1995), and family budgeting (Steinert et al., 2018), using a new digital delivery mechanism through an open-source offline-first mobile phone app. This is being tested in an ongoing cluster randomised trial of 4800 caregivers and adolescents in 80 sites.

Seventh, we conducted new methods to test discounted cost-effectiveness that allowed assessment of benefits across multiple outcomes (Grueso et al., 2024), and a new method to determine return on investment of accelerator policies and programmes. We developed a decision analytic model that combined three established economic models for health, education, and violence prevention. In doing so, it allowed estimation of cost-benefits across these domains, of the linkages and benefits between them, and of economic returns on investment in increased productivity.

We integrated economic and epidemiological models to evaluate the cost, impact, and productivity returns of three adolescent accelerators with strong evidence for their impact on multiple adolescent outcomes – cash transfers, parenting support, and adolescent-responsive sexual and reproductive health services. Comprehensive reviews of evidence on the impacts of these interventions and their implementation costs were utilised to parameterise established health impact and education models, as well as a Markov model for violence victimisation (Rudgard, Obiesie, et al. 2023). Gains in years of schooling were then translated into productivity returns, providing an estimate of the expected return that would be achieved from such an investment.

In order to support methodological capacity-sharing, we conducted training courses on the above approaches for researchers and government and UN partners across the region and made all code openly available on Github (here).

Each study in the review was conducted with institutional ethical approvals.

The 26 studies were gathered from 21 published manuscripts, 4 publications in progress, and 1 in press. All included studies were based in Africa, included adolescents according to the World Health Organization definitions, and include both protective domains and protective factors with clear measured outcomes. An abstraction sheet was created to list the study authors, year, and country, together with the sample characteristics (age, gender) listing protective domains and then protective factors and provisions. All outcomes were then listed according to the relevant provisions.

The second phase of evaluation was a broader lens of the entire accelerator hub initiative. The Hub was a large-scale initiative involving multiple partners. It included a range of academics, from English Literature through to Economics and Social Work, spread across universities in very different contexts. Activities included adolescent engagement and in-depth work with policy makers, in governments and international organisations. The Hub, structured in work packages, involving academics, adolescents and policy makers, was a form of collaborative inquiry (Lawrence et al., 2001). Different parties working together to learn about a common issue: how to identify appropriate accelerators for adolescents. In an effort to summarise our learning from the process, we undertook a reflective case study (Rittenhofer, 2015, p. 417). Reflective case studies typically involve in-depth interviews with participants in the case and review of documents. Here in place of the in-depth interviews, it takes the form of key participants co-authoring a summary of the key activities and lessons learned. This approach provides firsthand reflections, but runs the risk of bias, which we attempt to counter by providing evidence of our key points.

## Results

### Review of accelerator studies

**Figure 1.**
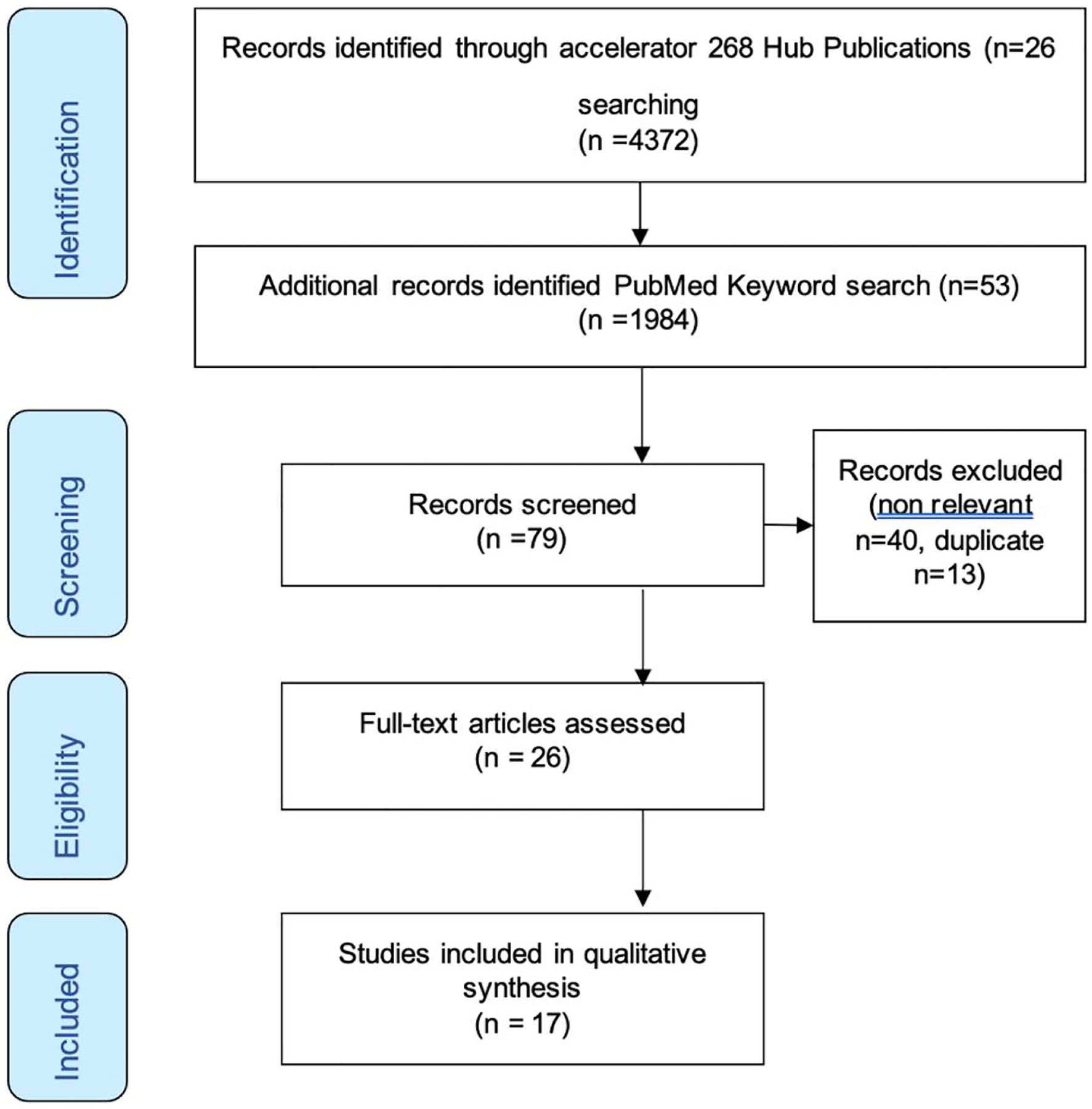
PRISMA diagram of search process.

Searches of all Accelerator Hub publications and PubMed is shown in a PRISMA diagram (Figure 1). We report on 26 papers across 10 countries – with some studies being multi-centred. Data was available from South Africa *n* = 16, Zambia *n* = 1, Malawi *n* = 2, Ghana *n* = 1, Ethiopia *n* = 1, Sierra Leone *n* = 1, Zimbabwe *n* = 2, Lesotho *n* = 1, Nigeria *n* = 1, Kenya *n* = 2, and Namibia *n* = 1, comprising information based on a total of 60,771 adolescents. Although not all studies disaggregated by gender, from those that provided this information, the data covered 40,243 females and 12,501 males. Abstracted data for the 26 studies are summarised in Table 1.

The 26 studies covered a wide range of protective domains (*n* = 11) including social protection, parenting/caregiver support, safe household/community, economic empowerment, education, psychological support, education, healthcare, norms and attitudes, caregiver support, childcare services. These covered a broad array of service-level provisions (*n* = 28) including cash transfers, parenting Support, safe schools, food security, positive parenting, parental monitoring and supervision, receiving praise from a caregiver, living in a safe community, community-based organisation access, mobile phone access, lifelong learning, living in abuse-free household, HIV prevention knowledge, emotional/social support, accessible (safe and affordable) healthcare, educational enrolment, cognitive stimulation, low student-teacher ratio, health extension programme, kind and respectful clinic access, safe schools, positive gender norms, healthy caregivers, no mental health issues, child not doing survival work, stable childhood, formal childcare, positive caregiver -adolescent relationship. Over the studies a variety of combinations or potential synergies of accelerators were tested (see table below)

### Emerging accelerators

**Figure 2.**
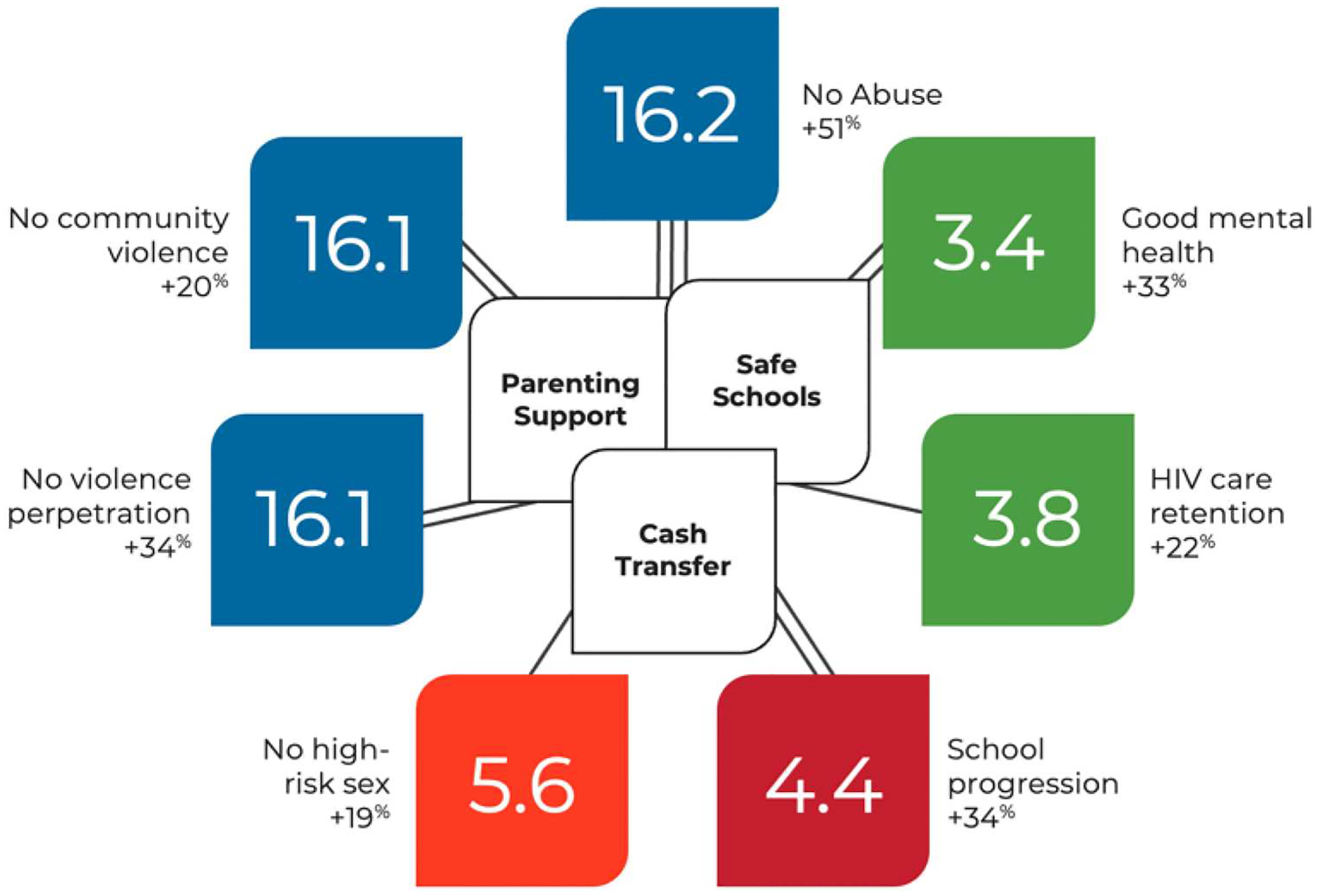
Three identified accelerators for SDG-aligned outcomes amongst adolescents living with HIV in South Africa. Single lines demonstrate that one of the accelerators showed an association with improvements in the outcome, double lines – two of the accelerators, and triple lines – three. Source: Cluver et al. (2019).

From the Figure 2, it can be seen, for example, amongst adolescents living with HIV in South Africa, three accelerators emerged: parenting support, government cash transfers, and safe schools. These had five, three, and four positive associations respectively among the eleven SDG target outcomes. In all, these three accelerators had beneficial associations with seven targets spanning four SDG goals (see Figure 1).

#### Cumulative effects:

Studies also tested whether cumulative effects were significant where more than one accelerator was associated with a given outcome, having a greater effect in combination than separately. This was termed a ‘synergy’ among the accelerators concerned. Simple combinations of two-to-three accelerators were associated with greater benefits on individual outcomes and with a wider range of SDG-aligned targets and goals.

Across the studies, the most common protective domain examined was social protection (appears in 38 elements), followed by parenting/caregiver support (20 elements), community safety (3 elements), education (3 elements), childcare services for adolescent mothers (2 elements), health care (3 elements), non-discriminatory gender norms/attitudes (3 elements), and one use of economic empowerment and psychological support. There is a wide range of outcome impacts, for both adolescent girls and boys, crossing the domains of risk, violence, mental health, education, lawbreaking, social interactions, and support seeking by adolescents and by caregivers. These are summarised in Table 2, which also sets out the accelerator and synergy accelerators described.

Social protection and food security were repeatedly identified as core accelerators. But they were not enough: psychosocial support, most commonly in the form of parental support (meaning support by any primary caregiver, whether a biological parent or not), was also fundamental. For example, in analyses of nationally representative datasets across Namibia, Lesotho, Mozambique, and Kenya, combined food security, parenting support, and positive gender norms were consistently and most strongly associated with reductions in HIV risks, adolescent pregnancy, child marriage, violence victimisation, mental health distress, sexual exploitation, and educational loss (Hertzog et al., 2024).

In contexts of high overall need, it was important to establish whether the most vulnerable young people benefited from similar or different accelerators. The research team also conducted primary research to develop new datasets with cohorts of in-school adolescents in parts of Nigeria and Ghana (Tamambang et al., 2024), amongst families with disabilities in Zambia (Chipanta et al., 2022), and with adolescent mothers in South Africa – a group comprising a third of all adolescent girls in the region, but with almost no focused policy or support services (Toska, Cluver, et al. 2020). In many cases, these found that the same accelerators (social protection and parenting support) produced even greater beneficial impacts on these highly vulnerable groups. In other cases, analyses identified responses specific to a defined vulnerable group. For example, provision of childcare services costing US$$9 per month increased adolescent mothers’ school enrolment, progression, future aspirations, and all forms of positive parenting and child stimulation, and childcare-attending children of adolescent mothers had a 27% increase in cognitive and motor development (Cluver et al., 2024).

Findings from the completed and ongoing randomised trials showed corresponding findings to those in the quasi-experimental analyses. In Kenya, cash transfers increased household revenue, assets, and consumption. When combined with psychosocial support – a motivational video and a long-term planning exercise – additional outcomes included reduced intimate partner violence and increased expenditure on girls’ and boys’ education (Orkin, 2022). In Tanzania: one-month post-intervention findings show reductions in sexual, physical, and verbal violence against adolescents and intimate partner violence against caregivers, improvements in positive parenting and caregiver and adolescent mental health, reductions in food insecurity, and improvements in household savings and gender-equitable norms and behaviours.

#### Additive benefits and optimum accelerators.

**Figure 3.**
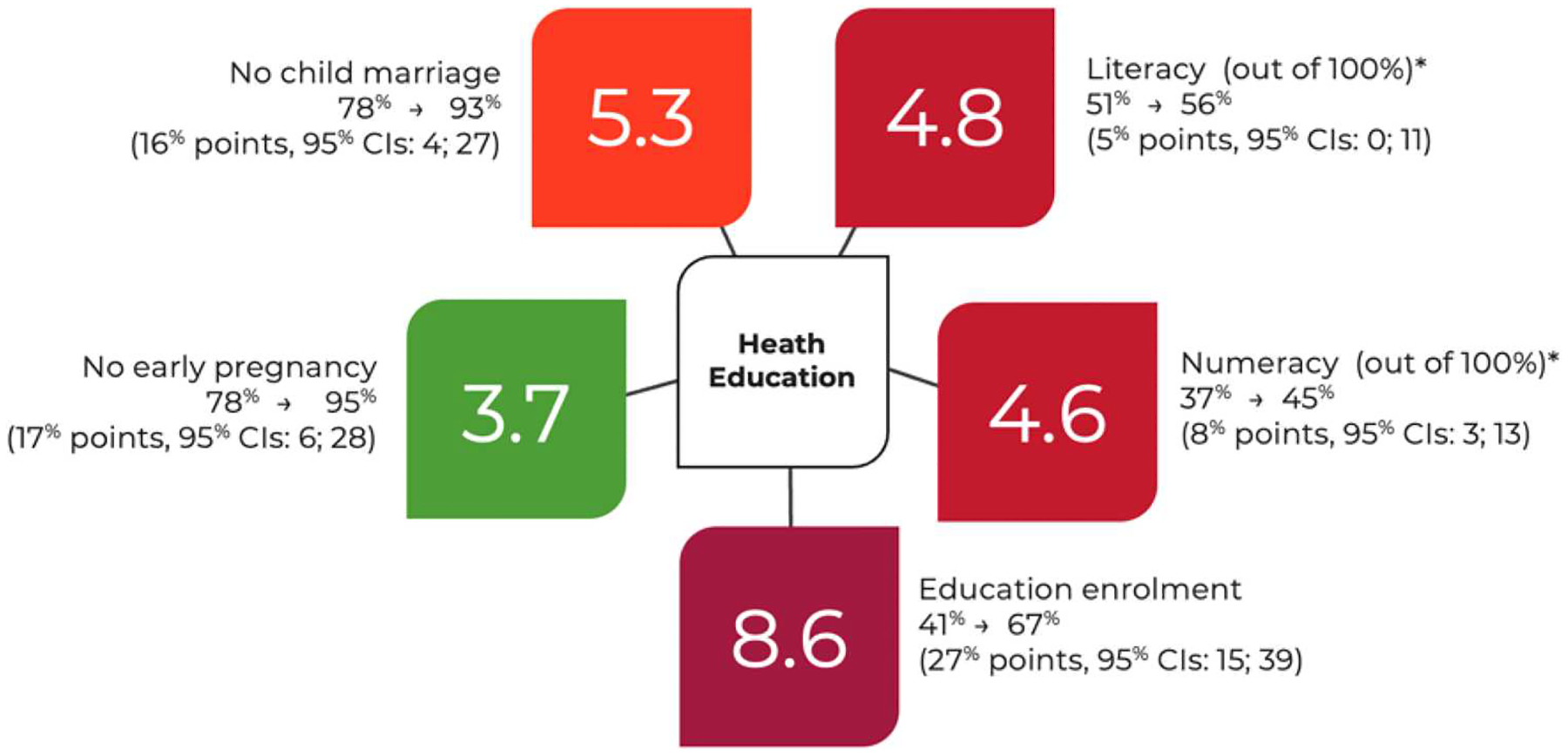
Rudgard, Dzumbunu et al., 2022, Multiple Impacts of Ethiopia’s Health Extension Program on Adolescent Health and Well-Being: A Quasi-Experimental Study 2002–2013. Journal of Adolescent Health. Source: Rudgard et al. (2022).

**Figure 4.**
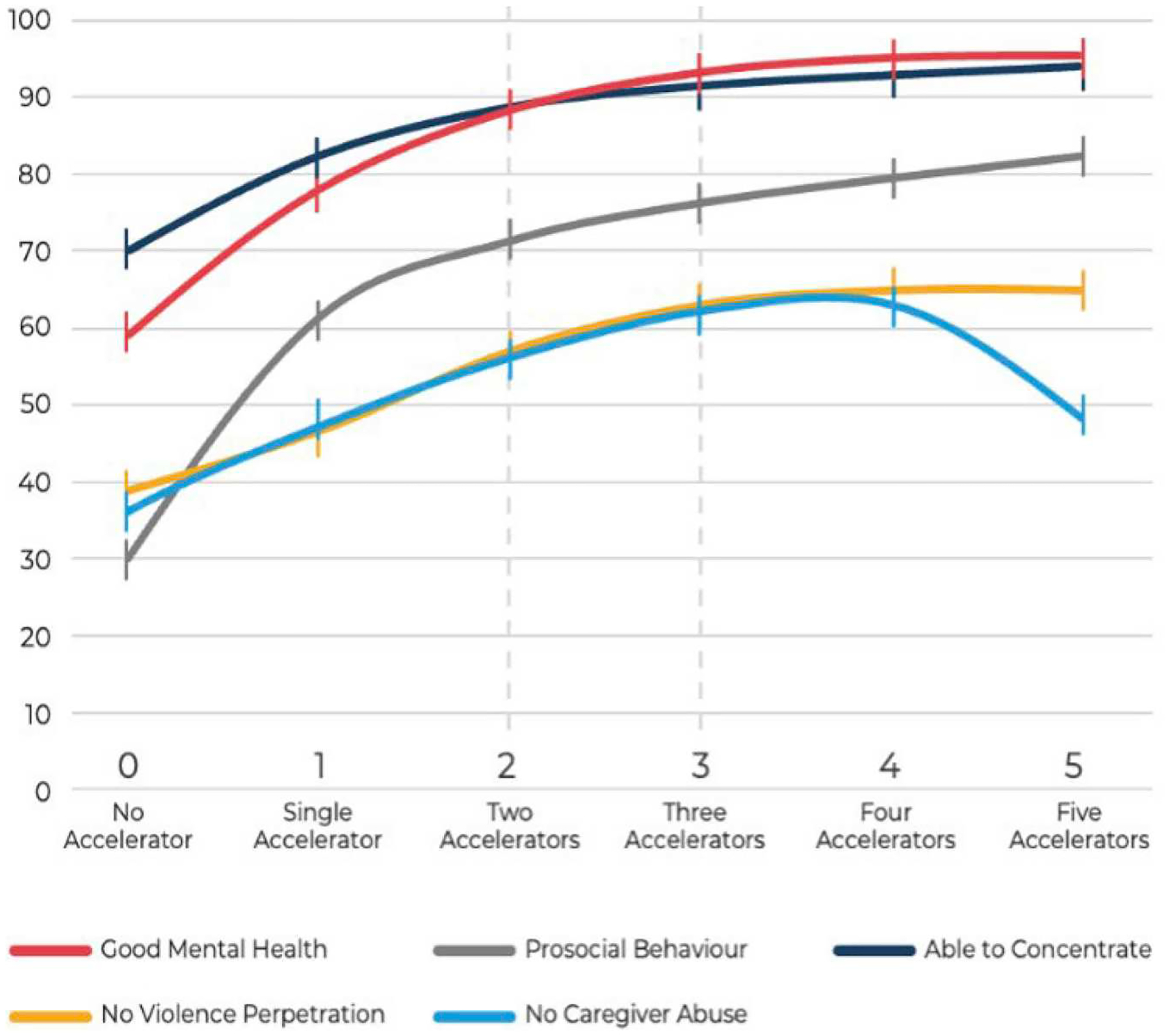
Associations of number of accelerator provisions with 16 adolescent outcomes in South Africa. Source: Sherr et al. (2023).

Within the 26 studies, one explored the additive benefits of layering accelerator provisions (Sherr et al., 2023). In South Africa. the maximum impact came from two-to-three accelerators, with impacts appearing to plateau after three (Figure 3 and Figure 4). This may help to focus and fine-tune investments to maximise impact and streamline cost. While meriting further testing, this observation recommends an implementation strategy in which interventions can be distributed in accordance with existing resources and provision for maximum effect.

Preliminary economic findings suggest that scaling-up a specific combination of accelerators is likely to be highly cost-effective, simultaneously impacting outcomes across health, education, and violence, while achieving a US$$ 4:1 return on investment in the future (Rudgard et al., 2024).

### Policy engagement and impact

This research aimed to have impact on policy for adolescents within Africa. An integrated policy and advocacy team provided support and initiative to operationalise findings into UN, government-level, and NGO programmes. This included training and support for African early-career researchers to include policy engagement in academic research, and also included responsive policy-relevant research.

Impacts include the study focused on adolescent mothers in South Africa, which brought together adolescent mothers in joint workshops with national policymakers to co-develop the government’s guidance for the new National Policy on the Prevention and Management of Learner Pregnancy in Schools (Jochim et al., 2023). In Lesotho, Eswatini, Cameroon, Kenya and Mozambique, the Global Fund to end AIDS, TB and Malaria used accelerator findings to directly inform guidance for national programs. Findings on returns on investment of accelerator interventions have been taken up by the Kenyan government and the World Bank, to inform a new $$50 m investment in adolescent girls, and by the Gates Foundation to inform child marriage prevention in Nigeria.

Adolescent-led advocacy groups have also utilised accelerator findings. For example, in Lesotho, HerVoice Fund civil society activists used this evidence to advocate for multi-component investments to reduce HIV acquisition risk in adolescent girls and young women in PEPFAR and Global Fund HIV programmes. In Sudan, we convened young civil society activists with bilateral and multilateral donors, to develop approaches for gender-transformative social protection for adolescents in conflict.

#### Responsive research in emergencies.

The COVID-19 pandemic required adaptation and new research to respond to increased risks for children and adolescents, and in doing so allowed assessment of whether accelerator interventions are feasible to deliver at scale in emergency settings. In all health emergencies, rates of violence against children increase. In March 2020, when 1.8 billion children had to leave their schools and enter extended global lockdowns, the research team initiated a rapid collaboration with UNICEF, the World Health Organization, the CDC, the Global Partnership to End Violence Against Children, and USAID (Cluver, Lachman, et al. 2020). This adapted evidence-based parenting programmes into a series of remote delivery options – tip sheets, public service announcements, social media messages, radio shows, and a song written and produced by a Broadway production team who volunteered support (Perks & Cluver, 2020). International teams of volunteers translated these into more than a hundred languages. Through the collaborative partners, and over 250 local NGOs, the resources reached over 210 million people worldwide and were used by 34 countries in their national COVID-19 responses (Sherr et al., 2022). In South Africa, the emerging evidence-base on the accelerator impacts of social protection allowed direct work with the Presidency to inform a social protection programme during the pandemic. This helped influence US$$6.8 billion in spending, and was found to have benefited 28.5 million people, preventing 5.5 million from falling into extreme poverty.

The research also responded to a global gap in epidemiological focus that unintentionally deprioritised children and adolescents. Pandemic reporting centred on deaths amongst adult populations, but there was no research focusing on children those deaths left behind. With the World Bank and CDC, the research team developed new techniques to merge rapidly-changing mortality and fertility data. This allowed the identification of more than 12 million children orphaned by COVID-19, two thirds of them adolescents (Hillis, Blenkinsop, et al., 2021, Hillis, Unwin, Chen, et al., 2021; Hillis et al., 2022; Unwin et al., 2022). This resulted in joint guidance with the World Health Organization, the World Bank, and the CDC (Hillis, Unwin, Cluver, et al., 2021), p. 1500 media articles, government policies to support COVID-19-associated orphans in Colombia and South Africa, a Papal decree, a White House Memorandum, and a new World Bank-led Rapid Social Response Fund in twelve countries. These examples suggest feasibility of delivery and policy uptake.

## Discussion

Adolescents are a fundamentally important and rapidly growing population group. Adolescence is an important developmental stage because of its implications over the life course: what we do or don’t do now to support adolescents will shape countries’ capacity to respond to future challenges. But in the current global context of multiple competing priorities, diminishing aid, and reduced prioritisation of social spending, it is essential to conceptualise and test alternative approaches to support.

This research aimed to identify and test accelerators for adolescents in Africa. It has found that the UN’s concept of development accelerators are highly effective and feasible to deliver. Findings show a strong case for investment, acceptability to adolescents and adaptability to local needs, strengths, provisions, and resources. These findings bring implications for policy and provision. Accelerators have the potential to allow providers to streamline resources, combine interventions to maximise impact, and support cross-departmental provision at govermment level. This has implications for integrated working, economic planning, costing, and human resources.

There remain structural challenges in operationalising accelerators. The UN Development Programme operationalises the concept through its ‘integrator’ function across the UN system. However, government departments or ministries have specific domain foci – such as education, health, justice – and often receive state funding in ways that do not incentivise investments in programmes that benefit multiple other ministries. Within international agencies and donors, these ‘silos’ are often replicated, with teams focused on specialised domains such as mental health, sexual health, or school enrolment for adolescent girls. These are each of fundamental importance, however, the accelerator approach provides a potential blueprint for harmonising them. We have identified existing and novel financing mechanisms that aim to address this mismatch between political incentives and logistical constraints (Desmond et al., 2023), but future work is needed to understand how to effectively promote inter-government collaboration and investment.

This body of work has limitations. Much of the evidence was interrogated from existing, quasi-experimental data sources, and there is now a need to expand the analysis in new randomised trials. The variables under scrutiny were limited to those measured in the studies conducted and reviewed, and other potential accelerators and outcomes may have been missed. The data was confined to specific countries and regional coverage may be needed.

Despite these considerations, there is clear evidence of the need and value of moving forward both the evidence-base and provision. Routine data collection must provide a more detailed account of adolescents, so that adolescent-specific provision can be mounted and monitored. Above all, both research and provision must put at their centre adolescents in their lived contexts. The future agenda must expand the evidence-base, incorporate a wider range of potential accelerators, and explore efficacy across a broader array of outcomes, guided at all stages by adolescent voices and priorities. The nature of accelerators – impacting on multiple domains simultaneously and with powerful interlinkages between them – necessitates research that is interdisciplinary, as a prerequisite for conceptual understanding, empirical analysis, and applied use. Accelerators are now included in several national and global policy documents, for example, by the World Health Organization, the World Food Programme, and UNICEF.

Even as we bring forward our understanding of high-impact investments for Africa’s adolescents, the continent is beginning to face a new set of challenges: climate change, social and political conflict, and epidemics, with Africa the most severely affected region (Atwoli et al., 2023). The Intergovernmental Panel on Climate Change (IPCC) forecasts increasing climate extremes over the next decade (Intergovernmental Panel on Climate Change, 2023). The World Health Organization warns of a next pandemic within this generation (World Health Organisation Report, 2023), and climate change may increase transmission of diseases such as cholera (Mora et al., 2022; Patton et al., 2016; Walika et al., 2023). For many adolescents in the region, these threats may overlap and exacerbate each other (Cappelli et al., 2023). As we look forward, we must sustain attention and responsive provision to adolescents even through protracted and overlapping challenges (Atwoli et al., 2023). By designing delivery within a paradigm of anticipated disruptions to basic services and recognition of holistic needs, we can prepare to protect adolescents through contexts of heightened risk.

The world faces a choice. We can continue as we are, with the inevitable risks for another generation, or we can develop new evidence and drive forward policy towards population-level, cost-effective accelerator services for adolescents.

## Figures and Tables

**Table 1.** Review of accelerators identified.

Author	Sample	Retained	Population	Age	Gender	Protective Domain	Protective factors/provisions	Outcome
1. Cluver et al. (2019) South Africa Cluver, L. D*., Or kin, F. M*., Campeau, L., Toska, E., Webb, D., Carlqvist, A., & Sherr, L. (2019). Improving lives by accelerating progress towards the UN Sustainable Development Goals for adolescents living with HIV: a prospective cohort study. The Lancet Child & Adolescent Health, 3(4), 245–254. (* Contributed equally)	1063	994 Female = 549 Male = 445	Adolescents living with HIV in the Eastern Cape province of South Africa	10–19 years	Male and Female	Social Protection	Cash Transfer	No Abuse School progression HIV care retention
						Social Protection	Parenting Support	No abuse No community violence No violence perpetration Good mental health No high-risk sex
						Social Protection	Safe School	No abuse No community violence No violence perpetration Good mental health School progression
2. Cluver, Rudgard, et al. (2022) South Africa Cluver, L.D*., Rudgard, W. E*., Toska, E., Or kin, M., Ibrahim, M., Langwenya, N.,… & Sherr, L. (2022). Food security reduces multiple HIV infection risks for high-vulnerability adolescent mothers and non-mothers in South Africa: a cross-sectional study. African Journal of Reproduction and Gynaecological Endoscopy, 25(8), e25928. (* Contributed equally)	1690	1690 1024 adolescent mothers 666 Non-mothers	AGYW without a live-born child (non-mothers), and AGYW who had conceived before age 20 and had a living child from that pregnancy (adolescent mothers).	11–25 years	Female	Social Protection	Food security	(Adolescent Girls) Lower odds of transactional sex Lower odds of multiple sex partners Lower odds of not being in education/employment
						Social Protection	Food security	(Adolescent mothers) Lower odds of transactional sex Lower odds of age disparate sex Lower odds of alcohol use Lower odds of sex on substances Lower odds of not being in education/employment
3. Cluver, Rudgard, et al. (2020) South Africa Cluver, L. D*, Rudgard, W. E*., Toska, E., Zhou, S., Campeau, L., Shenderovich, Y.,… & Sherr, L. (2020). Violence prevention accelerators for children and adolescents in South Africa: A path analysis using two pooled cohorts. PloS medicine, 17(11), e1003383. (* Contributed equally)	5034	4811 Female = 2087 Male = 2724	Adolescents living in selected areas of South Africa	10–19 years	Male and Female	Parenting support	Positive Parenting	(For Girls) Lower odds of sexual abuse Lower odds of physical abuse Lower odds of emotional abuse Lower odds of youth lawbreaking
						Parenting support	Parental Monitoring & Supervision	(For Girls) Lower odds of sexual abuse Lower odds of transactional sexual exploitation Lower odds of physical abuse Lower odds of emotional abuse Lower odds of community violence victimisation Lower odds of youth lawbreaking
						Social protection	Food Security	(For Girls) Lower odds of sexual abuse Lower odds of transactional sexual exploitation Lower odds of physical abuse Lower odds of emotional abuse Lower odds of community violence victimisation
						Parenting support	Positive Parenting	(For Boys) Lower odds of emotional abuse Lower odds of youth lawbreaking
						Parenting support	Parental Monitoring & Supervision	(For Boys) Lower odds of transactional sexual exploitation Lower odds of physical abuse Lower odds of emotional abuse Lower odds of community violence victimisation Lower odds of youth lawbreaking
						Social protection	Food Security	(For Boys) Lower odds of physical abuse Lower odds of emotional abuse
4. Haag et al. (2022) South Africa Haag, K., Du Toit, S., Rudgard, W. E., Skeen, S., Meinck, F., Gordon, S. L, … & Sherr, L. (2022). Accelerators for achieving the sustainable development goals in Sub-Saharan-African children and young adolescents–A longitudinal study. World Development, 151, 105739.	1848	1740 Female = 956 Male = 784	Children and adolescents living in South Africa	9–13 years	Male and Female	Social protection	Food security	No peer problems Right grade for age Able to concentrate at school Good mental health No MDD No caregiver abuse
						Safe community	Living in safe community	No MDD Good mental health No peer problems Higher prosocial behaviour No substance abuse Able to concentrate at school No early sexual debut No caregiver abuse
						Caregiver support	Receiving praise from a caregiver	No MDD Good mental health No suicidal ideation No PTSD No peer problems Prosocial behaviour No substance abuse No early sexual debut No violence perpetration
						Caregiver support	Consistent monitoring by a caregiver	No peer problems No early sexual debut No violence perpetration No PTSD Able to concentrate at school No caregiver abuse
						Social protection	CBO access	No MDD Good mental health No peer problems Higher prosocial behaviour No violence perpetration No peer problems
5. Chipanta et al. (2022) Zambia Chipanta, D., Estill, J., Stöckl, H., Hertzog, L, Toska, E., Chanda, *P*.,… & Cluver, L. (2022). Associations of sustainable development goals accelerators with adolescents’ well-being according to head-of-household’s disability status–A cross-sectional study from Zambia. International Journal of Public Health, 67, 1604341.	1,800	1725 Female = 881 Male = 844	Adolescents randomly sampled from households receiving social cash transfers.	16–24 years	Male and Female	Economic empowerment	Mobile phone access	No poverty Informal cash transfer Seeks mental health support No suicidal ideation School enrolment No health access restrictions related to disabilities Good health
						Social protection	Cash transfer	No poverty Informal cash transfer Seeks mental health support No suicidal ideation School enrolment No health access restrictions related to disabilities Good health
						Education	Lifelong learning	No poverty Informal cash transfer Seeks mental health support No suicidal ideation School enrolment No health access restrictions related to disabilities Good health
6. Mebrahtu et al. (2022) South Africa & Malawi Mebrahtu, H., Skeen, S., Rudgard, W. E., Du Toit, S., Haag, K., Roberts, K. J., …SSherr, L. (2022). Can a combination of interventions accelerate outcomes to deliver on the sustainable development goals for young children? Evidence from a longitudinal study in South Africa and Malawi. Child: Care, Health and Development, 48(3), 474–485.	989	854 Female = 446 Male = 408	Children and their caregivers affected by HIV and enrolled in community-based organisations	4–13 years	Male and Female	Social protection	Food security	School performance Learning progression Correct class for age No educational risk No cognitive delay
						Social protection	Cash grant	Correct class for age Not stunted No cognitive delay
						Safe community	Living in safe community	No depression symptomatology No trauma symptoms No mental health comorbidities
7. Toska, Campeau et al. (2020) Toska, E., Campeau, L, Cluver, L, Orkin, F. M., Berezin, M. N., Sherr, L, … & Bachman, G. (2020). Consistent provisions mitigate exposure to sexual risk and HIV among young adolescents in South Africa. AIDS and Behavior, 24, 903–913.	4811	3635 Female = 2048 Male = 1587	Adolescents living in two South African provinces,	10–17 years	Male and Female	Parenting support	Parenting/caregiver supervision	Reduced odds of exposure to sexual risk
						Safe household	Living in abuse-free homes	Reduced odds of exposure to sexual risk
						Social protection	Receiving at least one meal a day at school	Reduced odds of exposure to sexual risk
						Education	HIV prevention knowledge	Reduced odds of exposure to sexual risk
8. Du Toit et al. (2022) South Africa Du Toit, S., Haag, K., Skeen, S., Sherr, L, Orkin, M., Rudgard, W. E., …& Tomlinson, M. (2022). Accelerating progress towards improved mental health and healthy behaviours in adolescents living in adversity: findings from a longitudinal study in South Africa. Psychology, Health & Medicine, 27(sup1), 14–26.	333	290 Female 149 Male = 141	Adolescents	12–19 years	Male and Female	Social protection	Food security	No internalising behaviour Higher self-esteem No suicidal ideation
						Safe community	Safe environment	No substance abuse No internalising behaviour Healthy peer relationships
9. Hertzog et al. (2022) South Africa Hertzog, L, Banougnin, B. H., Stbckl, H., & Toska, E. (2022). Accelerating ontological security for South African adolescents living in high HIV-prevalence areas: a longitudinal study. Psychology, health & medicine, 27(sup1), 27–48.	1,519	1353 Female = 766 Male = 587	Adolescents living in HIV-prevalence areas	10–20 years	Male and Female	Social protection	Emotional and social support	Fewer symptoms of depression Fewer manifestation of anxiety Fewer attention deficit hyperactivity disorder Fewer negative future ideation Fewer exposure to sexual and emotional abuse Fewer domestic violence Fewer substance misuse.
						Parenting support	Parental/caregiver monitoring	Fewer symptoms of manifest anxiety Fewer PTSD Fewer negative future ideation Fewer physical and emotional abuse Fewer experience of community violence Fewer substance misuse
						Social protection	Accessible health care	Less symptoms of depression Less youth lawbreaking.
						Social protection	Food security	Fewer symptoms of manifest anxiety Fewer exposure to sexual abuse
10. Kusi-Mensah et al. (2022) Ghana Kusi-Mensah, K., Tamambang, R., Bella-Awusah, T., Ogunmola, S., Afolayan, A., Toska, E.,… Omigbodun, 0. (2022). Accelerating progress towards the sustainable development goals for adolescents in Ghana: a cross-sectional study. Psychology, Health & Medicine, 27(sup1), 49–66. https://doi.org/10.1080/13548506.2022.2108086	944	994 Female = 498 Male = 446	Adolescents	10y-19 years	Male and Female	Psychological support	Cognitive stimulation	School completion Access to ICT
						Education	Low student-teacher ratio	No open defecation Good mental health Access to ICT
						Social protection	No relative poverty	No open defecation School completion Access to ICT
11. Rudgard et al. (2022) Ethiopia Rudgard, W. E., Dzumbunu, S. *P*., Yates, R., Toska, E., Stbckl, H., Hertzog, L,… & Cluver, L. (2022). Multiple impacts of Ethiopia’s health extension program on adolescent health and well-being: A Quasi-experimental study 2002–2013. Journal of Adolescent Health, 71(3), 308–316.	850	775 Female = 355 Male = 420	Adolescents living in households supported by the HEP	15 years old	Male and Female	Social protection	Health extension program	(For Boys) Higher odds of education enrolment Higher literacy Lower odds of >4 h in income-generating activities per day
						Social protection	Health extension program	(For Girls) Higher odds of no adolescent pregnancy Higher education enrolment Higher odds of no child marriage Higher numeracy Higher literacy
12. Sherr et al. (2021) South Africa & Malawi Sherr, L, Roberts, K.J., Tomlinson, M. et al. Food Should not be Forgotten: Impacts of Combined Cash Transfer Receipt and Food Security on Child Education and Cognition in South Africa and Malawi. AIDS Behav 25, 2886–2897 (2021). https://doi.org/10.1007/Si0461-021-03317-6	854	796 Female = 416 Male = 380	Children and families accessing community-based organisations (CBOs)	5–15 years	Male and Female	Social protection	Food security	Reduced educational risk scores Greater odds of being in the correct class for age, Greater odds of regular school attendance Greater odds of missing less than a week of school in two weeks
						Social protection	Cash grant	Reduced educational risk scores Greater odds of being in the correct class for age, Greater odds of regular school attendance Greater odds of missing less than a week of school in two weeks
13. Sherr et al. (2016) South Africa Sherr, L, Yakubovich, A. R., Skeen, S., Cluver, L. D., Hensels, 1. S., Macedo, A., & Tomlinson, M. (2016). How effective is help on the doorstep? A longitudinal evaluation of community-based organisation support. PLoS One, 11(3), e0151305.	1848	1741 CCC = 383 YC = 1358	Adolescents	9–13 years	Male and Female	Social protection	CBO support services	Lower odds of domestic violence Lower odds of abuse Lower odds of suicidal ideation Fewer depressive symptoms Less perceived stigma Fewer peer problems Fewer conduct problems More prosocial behaviour
14. Dunbar et al. (2014) Zimbabwe Dunbar, M. S., Kang Dufour, M. S., Lambdin, B., Mudekunye-Mahaka, 1., Nhamo, D., & Padian, N. S. (2014). The SHAZ! project: results from a pilot randomized trial of a structural intervention to prevent HIV among adolescent women in Zimbabwe. PloS one, 9(11), e113621.	315	315 Intervention group = 158 Control group = 157	Adolescent female orphans (having lost at least one parent)	16–19 years	Female	Social protection	SHAZI intervention	Reduced food insecurity Earn income Reduced transactional sex Increase condom use Reduced violence
15. Cluver et al., South Africa (2025) Cluver, L. D.,* Jochim, J.,* (equal first), Banougnin, B. H., Roberts, K. J., de Graaf, K., Toska, E. (* Contributed equally)	1044	771	Adolescent mothers	10–24 years	Female	Social protection	Food security	Reduced transactional/age disparate sex Reduced sex without contraception; Increased school enrolment or work engagement; Reduced low self-efficacy
						Parenting support	Non-violent parenting	Reduced suicidality Reduced mental ill-health Reduced transactional/age disparate sex Reduced intimate partner violence Reduced sexual violence.
						Healthcare	Respectful clinic access	Reduced mental ill-health Reduced low self-efficacy Reduced condomless sex.
16. Hertzog et al. (2024) Lesotho Hertzog, L.,* Cluver, L. D.,* Banougnin, B. H., Saminathen, M. G., Little, M. T., Mchenga, M., Yates, R., Rudgard, W. E., Chiang, L., Annor, F., Picchetti, V., Massetti, G., Hegle, J., Foraci, M., Linko, M., Toska, E. (2024). Social protection as a strategy for HIV prevention, education promotion and child marriage reduction among adolescents: a cross-sectional population-based study in Lesotho. BMC Public Health, 24. (* Contributed equally)	3,506	3506 Female =2,881 Male = 625	Adolescents and young people living in poverty	13–24 years	Male and Female	Social protection	Social protection service	(For Girls) Reduced child marriage Increased consistent condom use School enrolment Engaged in any paid work (Sub-group of girls and young women over 18)
						Social protection	Social protection service	(For Boys) School enrolment Educational attainment
17. Little et al., Namibia (2025) Little, M. T.,* Hertzog, L, Rudgard, W. E., Toska, E., Banougnin, B. H., Yates, R., Chipanta, D., Annor, F., Laura Chiang, L.,& Cluver, L. D. (In view) (* Corresponding author)	5167	5167 Female = 4189 Male = 978	Adolescents and young people represented in the 2019 Namibia VACS	13–24 years	Males & Female	Social protection	Food security	(For Girls) Increase in safe sex Reduced early sex debut Reduced tobacco use Good mental health
						Parenting support	Safe parenting	(For Girls) Good mental health Reduced online harassment Reduced physical fight Community safety Reduced physical/emotional IPV Reduced sexual violence Reduced transactional sex
						Social protection	Safe school	(For Girls) Reduced age disparate sex Reduced physical/emotional IPV Reduced sexual violence Reduced transactional sex
						Parenting support	Safe parenting	(For Boys) Increased safe sex Reduced online harassment Reduced physical fight Community safety Reduced physical/emotional IPV Good mental health
						Norms & attitude	Positive gender norms	(For Boys) Reduced multiple sexual partners Reduced heavy alcohol use Reduced transactional sex Reduced sexual violence Reduced physical/emotional IPV Community safety
18. Meinck et al. (2021) South Africa Meinck, F., Orkin, M., & Cluver, L. (2021). Accelerating Sustainable Development Goals for South African adolescents from high HIV prevalence areas: a longitudinal path analysis. BMC medicine, 19, 1–17.	3516	3401 Female = 1926 Male = 1475	Adolescents in areas with >30% HIV prevalence	10 – 17 years	Males & Female	Parenting support	Positive parenting	Less physical abuse Less emotional abuse Less substance use Less suicidal ideation
						Parenting support	Parental monitoring	Less physical abuse Less emotional abuse Less HIV risk behaviour Less bullying Less substance use Less experience of community violence
						Social protection	Food sufficiency	Less physical abuse Less emotional abuse Less witness of domestic violence Less suicidal ideation Less bullying Less witness of community violence Less experience of community violence Less HIV risk behaviour
						Caregiver support	AIDS-free caregiver	Less physical abuse Less emotional abuse Less experience of community violence Less suicidal ideation Less bullying Less witness of domestic violence Less TB symptomatology Less HIV risk behaviour
19. Tamambang et al. (2024) Nigeria Tamambang, R., Kusi-Mensah, K., Bella-Awusah, T., Ogunmola, 0., Afolayan, A., Toska, E.,… Omigbodun, 0. (2024). Identifying potential catalysts to accelerate the achievement of Sustainable Development Goals (SDGs) among adolescents living in Nigeria. Psychology, Health & Medicine, 29(4), 868–887. https://doi.org/10.1080/13548506.2023.2289476	1800	1800	Adolescents in Southwest Nigeria	10–19 years	Males & Female	Social Protection	Food security	More likely not to use psychoactive substances More likely to have normal school progression More likely to have positive protection under child rights (no risky sexual behaviour, no self-perpetration of violence, no community violence, and no sexual abuse).
						Health and Wellbeing	No mental health issues	Less likely to use substances More likely to have good educational outcomes More likely to have positive protection under child rights (no risky sexual behaviour, no self-perpetration of violence, no community violence, and no sexual abuse)
						Social protection	Safe schools	Able to concentrate in school. Optimal school attendance No self-perpetration of violence. No community violence. No sexual abuse Not engaging in substance use.
						Social protection	Child not doing survival work	Have normal BMI Not being stunted Progressing well in school.
						Social protection	Stable childhood	Not being stunted Not engaging in substance use. Progressing well in school.
						Parenting support	Parental support	No use substances Optimal school attendance No risky sexual behaviours
20. Toska et al. (2024) South Africa Toska, E., Zhou, S., Laurenzi, C. A., Saal, W., Rudgard, W., Wittesaele, C, …& CIuver, L. (2024). Healthcare provisions associated with multiple HIV-related outcomes among adolescent girls and young women living with HIV in South Africa: a cross-sectional study. Journal of the International AIDS Society, 27(2), e26212.	774	774	AGYW living with HIV	12–24 years	Female	Healthcare provision	Kind and respectful healthcare providers	Past-week adherence Consistent clinic attendance Uninterrupted ART treatment Viral suppression
						Healthcare provision	Safe and affordable facilities	Consistent clinic attendance Uninterrupted treatment No TB symptoms
21. Banougnin et al. (2023) South Africa Cluver, L.D* Jochim, J*., Mapukata, Y., Wittesaele, C, Shenderovich, Y., Mafuya, S., Steventon Roberts, K., Banougnin, B., Sherr, L, & Toska, E. (2023). Associations of formal childcare use with health and human capital development for adolescent mothers and their children in South Africa: A cross-sectional study. Child: Care, Health and Development, 50(1), e13138. https://doi.org/10.1111/cch.13138 (* Contributed equally)	Adolescent mothers = 1046 Children = 1139	Adolescent mothers = 776 Children = 837	Adolescent mothers and their children	10–19 years	Female	Childcare service	Formal childcare (For mothers)	Higher odds of being enrolled in secondary school Higher odds of being enrolled in tertiary education Higher odds of being engaged in employment Higher odds of grade promotion Higher odds for positive future ideation Higher levels of positive parenting Better parental limit-setting Better positive discipline
						Childcare service	Formal childcare (For their children)	Better cognitive, language and motor development with increasing child age
22. Rudgard, Saminathen, et al. (2023) South Africa Rudgard, W. E., Saminathen, M. G., Orkin, M., Banougnin, B. H., Shenderovich, Y., & Toska, E. (2023). Protective factors for adolescent sexual risk behaviours and experiences linked to HIV infection in South Africa: a three-wave longitudinal analysis of caregiving, education, food security, and social protection. BMC Public Health, 23(1), 1452.	1563	1563 (70% living with HIV) Girls = 906 Boys = 657	Adolescents living with HIV and not living with HIV	10–19 years	Male and Female	Parenting support	Positive parenting	For Girls Lower probability of multiple HIV risk behaviours Lower probability of transactional sexFor Boys
								Lower probability of transactional sex Higher probability of condomless sex
						Parenting support	Caregiver supervision	For Girls Lower probability of multiple HIV risk behaviours Lower probability of transactional sex Lower probability of age-disparate sex Lower odds of subsequently experiencing multiple sexual partners Lower odds of condomless sex Lower odds of sex on substancesFor Boys
								Lower probability of multiple sexual partners Lower probability of transactional sex Lower probability of age-disparate sex Lower probability of sex on substance
						Social protection	Food secure	For Girls Lower probability of multiple sexual partners Lower probability of transactional sex Lower odds of subsequently experiencing multiple sexual partners Lower odds of condomless sexFor Boys
								Lower probability of transactional sex
						Social protection	Education enrolment	For Girls Lower probability of age-disparate sex Lower probability of condomless sex
23. Banougnin et al. (2023) South Africa Banougnin, B. H., Toska, E., Maughan-Brown, B., Rudgard, W., Hertzog, L, Jochim, J.,… Cluver, L. (2023). Associations of social media and health content use with sexual risk behaviours among adolescents in South Africa. Sexual and Reproductive Health Matters, 31(4). https://doi.org/10.1080/26410397.2023.2267893	1563	1410 (69% living with HIV)	adolescents living with and without HIV	10–24 years	Male and Female	Health provision	Informal m-Health	Lower rates of sex after substance use Lower rates of unprotected sex.
24. Maughan-Brown et al. (2025), Kenya Maughan-Brown, B., Banougnin, B. H., Little, M. T., Hertzog, L, Matsha-Carpentier, N., Mugambi, C, Gichane, H., Cluver, L. D., Toska, E. Tackling the Triple Threat in Kenya: Factors Associated with Protection against HIV Risk, Gender-Based Violence, and Pregnancy among Adolescent Girls and Young Women. AIDS and Behavior, https://doi.org/10.1007/s10461-025-04643-9	1344	1344	Adolescent girls and young women	13–24 years	Female	Gender norms	Gender-equitable attitudes	Lower probabilities of IPV Lower probabilities of adolescent pregnancy
						Social protection	Food security	Less likely to report adolescent pregnancy Less likely to report child marriage
						Parenting support	Parental support	Less likely to report IPV Less likely to report sexual violence Less likely to report adolescent pregnancy Less likely to report child marriage
25. Mchenga et al., Zimbabwe (2025)	7211	7211	AGYW (53% were within the age ranges 18–24)	13–24 years	Female	Parenting support	Positive caregiver-adolescent relationship	Reduced odds of experience of any form of sexual abuse in the past year Reduced odds of physical IPV in the past year Reduced odds of child abuse (emotional or physical) in the past year Reduced odds of alcohol consumption in the past 30 days Reduced odds of mental distress in the past 30 days Reduced odds of early sexual debut
						Social protection	Food security	Lower odds of physical intimate partner violence in the past Lower odds of peer physical violence in the past year Lower odds of child abuse (emotional or physical) in the past year Lower odds of mental distress in the past 30 days Lower odds of adolescent pregnancy
						Gender norms	Gender-equitable attitudes	Reduced odds of inconsistent condom use in the past year Reduced odds of age-disparate sex Reduced odds of any sexual abuse in the past year Reduced odds of physical IPV in the past year Reduced odds of child abuse (physical or emotional) in the past year Reduced odds of not in school or paid work in the past year Reduced odds of mental distress in the past 30 days Reduced odds of early sexual debut before the age of 16 Reduced odds of child marriage before theageof18 Reduced odds of adolescent oreanancv

**Table 2.** Accelerators and Synergy accelerators across countries.

Accelerator	Synergy accelerators	Country	Common combinations
Cash transfer	Food security	Malawi	Not stunted
	Safe community		No cognitive delay
	Safe school	South Africa	Correct class for age
	Food security		Regular school attendance
	Safe community		
	Parenting support		
	Lifelong learning	Zambia	
	Mobile phone access		
Food security	Cash transfer	Malawi	Reduced educational risk scores
	Safe community		Good mental health
	Cash transfer	South Africa	Correct class for age
	Safe community		Regular school attendance
	Safe parenting		Missing less than a week of school in two weeks
	Accessible healthcare		
	Respectful clinic access		
	Emotional and social support		
	Parental/caregiver monitoring		
	Safe school	Namibia	
	Safe parenting		
	Positive gender norms		
Safe community	Food security	Malawi	No depression symptomatology
	Cash transfer		No trauma symptoms
	CBO access	South Africa	No mental health comorbidities
	Cash transfer		
	Food security		
	Receiving praise from a caregiver		
	Consistent monitoring by a		
	caregiver		
Safe parenting	Safe school	Namibia	Good mental health
	Food security		Reduced sexual violence & abuse
	Positive gender norms		Safety in community
	Food security	South Africa	Reduced transactional sex
	Respectful clinic access		
